# Morbidity Prevalence Estimate at 6 Months Following a Stroke: Protocol for a Cohort Study

**DOI:** 10.2196/15851

**Published:** 2020-06-17

**Authors:** Alexander Smith, Natalie Bains, Lauren Copeland, Anna Pennington, Ben Carter, Jonathan Hewitt

**Affiliations:** 1 Division of Population Medicine Cardiff University Cardiff United Kingdom; 2 Research and Development Aneurin Bevan University Health Board Newport United Kingdom; 3 Department of Biostatistics and Health Informatics Institute of Psychiatry, Psychology & Neuroscience King’s College London United Kingdom; 4 Aneurin Bevan University Health Board Ysbyty Ystrad Fawr Ystrad Mynach United Kingdom

**Keywords:** stroke, prevalence estimate, morbidity, disability, PROMs, outcomes, quality of life (QoL)

## Abstract

**Background:**

Knowledge of the prevalence of morbidity secondary to stroke is important for health care professionals, health care commissioners, third sector organizations, and stroke survivors to understand the likely progress of poststroke sequelae and to aid in commissioning decisions, planning care, and adjusting to life after stroke.

**Objective:**

The primary aim of the Morbidity PRevalence Estimate In StrokE (MORe PREcISE) study is to determine the prevalence of morbidity secondary to a stroke, predictors of morbidity, and trends in quality of life and functional status using patient-reported outcomes, cognitive and functional assessments.

**Methods:**

A total of 500 participants will be recruited across Wales and England within 14 days following an admission to a stroke unit for either an ischemic or hemorrhagic stroke as part of a multicenter cohort study. Participants are assessed at baseline ≤14 days poststroke and subsequently at 90 (± 14) days and 180 (± 14) days poststroke. At each time point, data will be collected relating to the following domains: participant demographics, routine clinical, patient reported, cognitive status, emotional well-being, and functional ability.

**Results:**

Recruitment commenced in October 2018 with 20 sites opened as of September 2019 and was closed on October 31, 2019.

**Conclusions:**

The primary outcome is the prevalence of morbidity at 6 months secondary to a stroke. Further analysis will consider temporal changes in the health-related domains to describe trends among baseline, 3-, and 6-month time points.

**Trial Registration:**

ClinicalTrials.gov NCT03605381; https://clinicaltrials.gov/ct2/show/NCT03605381

**International Registered Report Identifier (IRRID):**

DERR1-10.2196/15851

## Introduction

### Stroke Morbidity

There are 1.2 million stroke survivors currently living in the United Kingdom [1]. Although mortality as a consequence of stroke is decreasing [1,2], over two-thirds of stroke survivors have a form of disability on discharge from hospital [3]. During a stroke, hypoxic injury leads to neuronal death [4,5], which can occur in potentially any part of the brain. Thus, due to the diverse functions of the brain, stroke can lead to significant impairments to diverse functions and structures of the body, resulting in a significant prevalence of morbidity secondary to stroke [6].

The prevalence of morbidity secondary to stroke is of central importance to health professionals to understand the prognosis of the disease in patients under their care. Further, providing an accurate estimation of the prevalence of morbidity secondary to stroke will allow commissioners of care, planners, and third sector organizations to adapt to and answer the needs of a poststroke population. Additionally, information regarding the likely progression of impairments secondary to stroke is important to stroke survivors, allowing them to plan for the future and to adjust to life after stroke.

### Measures of Morbidity

Expanding data collection beyond current routinely collected data in stroke relates to the work undertaken by the International Consortium for Health Outcomes Measurement (ICHOM) [7] and their minimum outcome dataset for stroke [8]. In the ICHOM Standard Set for Stroke, a number of, what ICHOM terms, “variables” (demographic, clinical, and treatment) and “outcomes” (survival, disease control, and patient reported) are included. The ICHOM Standard Set for Stroke takes important steps to collect data outside of the process of care data in areas such as patient-reported outcome data, which includes domains such as toileting, walking, and assistance with feeding. However, the ICHOM group does not advocate the specific collection of data related to cognitive impairment or emotional problems secondary to stroke. This study will address this by the inclusion of measures of emotional problems and mild cognitive impairment. Therefore, the scope of this study is to build on routinely collected health care and poststroke data not currently collected by the ICHOM Standard Set for Stroke for the purpose of estimating morbidity prevalence.

### Aims and Objectives

This paper describes the protocol for the Morbidity Prevalence Estimate at 6 Months Following a Stroke (MORe PREcISE) study (ClinicalTrials.gov NCT03605381—Registered: 30/07/19). The primary objective is to determine the prevalence of morbidity at 6 months secondary to a stroke, predictors of morbidity, trends in health-related quality of life (HRQoL), and function. The definition of morbidity secondary to stroke includes the following: aphasia, anxiety, depression, dysarthria, dysphagia, hemianopia, hemiparesis, hemiplegia, hemi-inattention, cognitive impairment, and functional impairment including activities of daily living and social interaction or roles.

Therefore, this study will collect a wide range of data related to the most common morbidity secondary to stroke, from stroke onset to 6 months poststroke across England and Wales. The secondary objectives include describing trends in domains such as HRQoL from acute onset to 6 months poststroke. It will also explore trends in pre- and poststroke functional levels, as assessed by the Modified Rankin Scale (mRS) [9] from the prestroke period to 6 months poststroke. Lastly, trends in outcomes (patient reported, functional, treatment, and process of care) by geographical distribution, such as country, health board/trust, local authority, and hospital (research site) coverage, will be explored.

## Methods

### Study Design

This study uses a 6-month prospective cohort study of stroke survivors and aims to recruit 500 participants between August 2018 and October 2019. Data measuring morbidity will be collected at three distinct periods: baseline (≤14 days poststroke), 3 months, and 6 months poststroke. This takes place in 20 centers across England and Wales which routinely admit acute or hyperacute stroke patients (Textbox 1). All sites were selected after expressing an interest via the National Health Service (NHS) research networks, and the geographic spread was considered during site selection.

**Participating research sites.**

Participants:

- Aneurin Bevan University Health Board, South East Wales, United Kingdom
- Bronglais General Hospital, Aberystwyth, Wales, United Kingdom
- Glangwili General Hospital, Carmarthen, Wales, United Kingdom
- Gloucestershire Royal Hospital, Gloucester, England, United Kingdom
- Kingston Hospital, London, England, United Kingdom
- University Hospital of Wales, Cardiff, Wales, United Kingdom
- Morriston Hospital, Swansea, Wales, United Kingdom
- New Cross Hospital, Wolverhampton, England, United Kingdom
- Oxford University Hospitals, Oxford, England, United Kingdom
- Peterborough City Hospital, Peterborough, England, United Kingdom
- Prince Charles Hospital, Merthyr Tydfil, Wales, United Kingdom
- Princess of Wales Hospital, Bridgend, Wales, United Kingdom
- Somerset Partnership (SOMPAR), Somerset, England, United Kingdom
- Southmead Hospital, Bristol, England, United Kingdom
- University Hospital Lewisham, London, England, United Kingdom
- West Middlesex University Hospital, London, England, United Kingdom
- Withybush General Hospital, Haverfordwest, Wales, United Kingdom
- Wrexham Maelor Hospital, Wrexham, United Kingdom
- Yeovil District Hospital, Yeovil, England, United Kingdom
- Ysbyty Glan Clwyd, Bodelwyddan, Wales, United Kingdom

### Participants and Eligibility Criteria

Participants eligible to be recruited for this study include those aged 18 years or over with a clinical diagnosis of stroke, within the previous 14 days; cerebral infarct (ICD I63) [10]; intracerebral hemorrhage (ICD I61) [10]; or stroke not specified as hemorrhagic or infarction (ICD I64) [10]. Exclusion criteria include a diagnosis of transient ischemic attack (ICD G45) [10], subarachnoid hemorrhage (ICD I60) [10], or any condition defined under ICD G93 (eg, anoxic brain damage) [10]. Patients receiving palliative care or are eligible for palliative care are also excluded from this study.

#### Sample Size

In order to estimate the prevalence of stroke morbidity at 6 months, assuming a 35% morbidity rate, with a 95% CI width of ±5%, will require 350 stroke survivors. Assuming that there is a 30% dropout [11] at the 6-month visit, the aim is to recruit a minimum of 500 stroke survivors. This will be achieved by aiming to approach all appropriate inpatients fulfilling the eligibility criteria across the 20 sites. Stata Statistical software will be used for the analyses.

### Outcomes

The primary outcome is to quantify the prevalence of poststroke morbidity, at three time points, using a range of assessments and outcome measures, which are as follows:

- Patient-reported outcomes:
  - Patient-Reported Outcomes Measurement Information System Global Health Short Form-10 (PROMIS-10) [12].
  - Three Questions from the Riksstroke [13].
  - Two Questions from the ICHOM Standard Stroke Set for Stroke [8].
- Cognitive status:
  - Short-Form Montreal Cognitive Assessment (SF-MoCA) [14] (domains, clock drawing, abstraction, five-word recall).
  - Telephone Montreal Cognitive Assessment Short (T-MoCA-Short) [15] (domains, verbal fluency, orientation, five-word recall).
- Emotional well-being:
  - Patient Health Questionnaire-4 (PHQ-4) [16].
  - PHQ-9 [17].
  - Generalized Anxiety Disorder-7 (GAD-7) [18].
- Functional ability:
  - Modified Rankin Scale [9].
  - Rankin Focused Assessment (RFA) [19].
- Treatment:
  - Recurring stroke (cerebral infarct [ICD I63]), intracerebral hemorrhage [ICD I61], and stroke not specified as hemorrhage or infarction [ICD I64] [10].
- Process:
  - Length of stay following primary admission for stroke.
  - Readmission to hospital within 30 days of discharge.

### Data Collection

**Figure 1:**
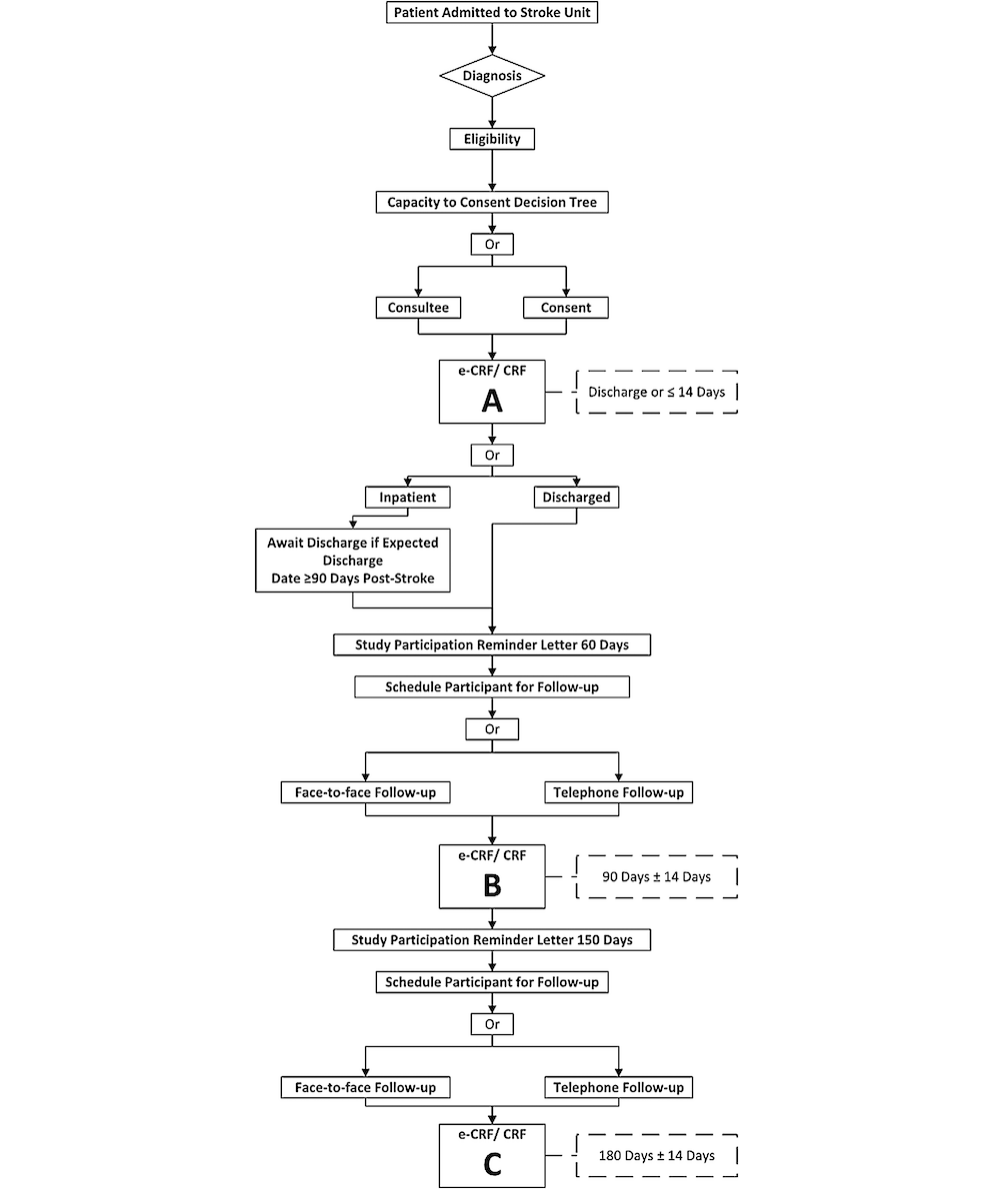
Participant flow diagram. Electronic Case Report Form/Case Report Form (eCRF/CRF).

**Figure 2:**
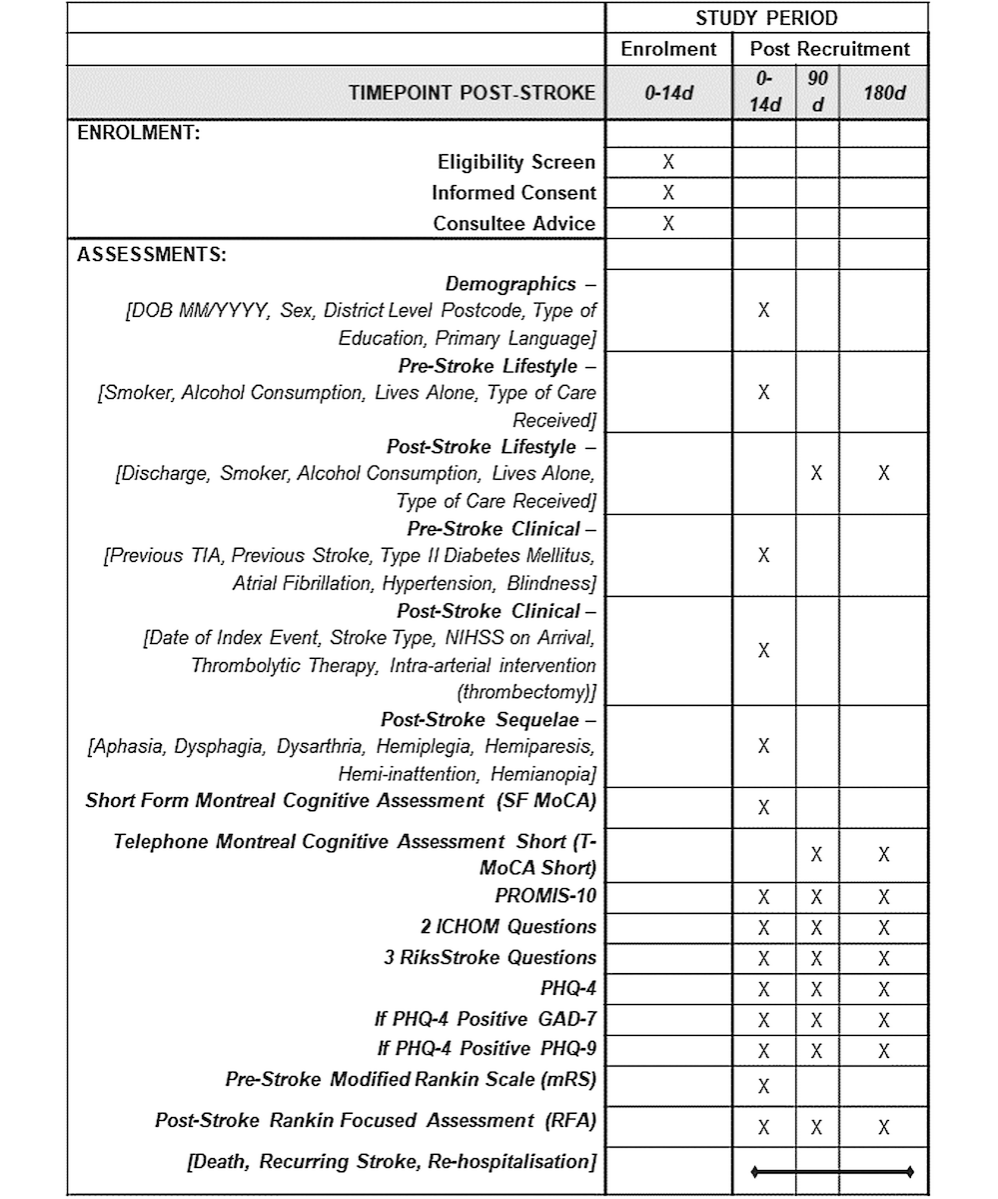
SPIRIT (Standard Protocol Items: Recommendations for Interventional Trials) checklist. SF MoCA: Short-Form Montreal Cognitive Assessment; T-MoCA-Short: Telephone Montreal Cognitive Assessment Short; PROMIS-10: Patient-Reported Outcomes Measurement Information System Global Health Short Form-10; ICHOM: International Consortium Health Outcomes Measurement; PHQ-4: Patient Health Questionnaire-4; GAD-7: Generalized Anxiety Disorder-7; PHQ-9: Patient Health Questionnaire-9; mRS: Modified Rankin Scale; RFA: Rankin Focused Assessment.

Following enrolment into the study, data collection is divided into three distinct periods: Period A (within 14 days from the onset of stroke), Period B (90 days poststroke ± 14 days), and Period C (180 days poststroke ± 14 days) as per the participant flow diagram (Figure 1). A standardized SPIRIT (Standard Protocol Items: Recommendations for Interventional Trials) [20] schedule of assessments is presented in Figure 2.

#### Period A—Within 14 Days of Stroke Onset

Period A begins following consent or following consultee declaration with data collection attempted as soon as possible during the participant’s admission. Baseline data collection will occur at 14 days or less poststroke and before discharge from hospital. All data will be collected using Case Report Form A (CRF A). There is a further window of 24 hours available for data collection if necessary. Baseline data will not be recorded outside these time points. Additionally, provided consent was received before discharge, data collection from the participant may occur after discharge to the community if no opportunity arose prior to discharge.

The baseline data collected comprise participant demographic and routine clinical data including their prestroke lifestyle data, cognitive assessment, Patient Reported Outcome Measure (PROM), and anxiety and depression screen. Demographic data are collected via medical notes or a care team and then confirmed by the participant or participant’s family or friends. An equivalent process will be undertaken to gather prestroke lifestyle data.

During baseline data collection, a cognitive screen will be administrated in the form of a SF-MoCA [14]. Alongside this assessment, the participants are required to self-complete a PROM using the PROMIS-10 [12], Riksstroke questions [13], and ICHOM questions [8]. Participants will also self-complete a PHQ-4 [16]. If the PHQ-4 is positive, as indicated by a score ≥3 for the sum of questions 1 and 2 or 3 and 4, for anxiety or depression, the patient will then self-complete the PHQ-9 [17] and GAD-7 [18]. If the participants are unable to self-complete, the study allows for assessments or outcomes to be administered verbally.

To gain a representation of any prestroke disability in participants, a prestroke mRS [9] will be completed. This must be carried out by a suitably trained health care professional. RFA [19] will be carried out by a suitably trained health care professional for an estimate of the level of poststroke disability.

#### 60 Days Poststroke (± 7 Days)

Between Periods A and B at 60 days poststroke, it should be established whether the participant remains as an inpatient or is discharged. It should be ascertained whether the participant has deceased, had another stroke, or is eligible for or already receiving palliative care. In the case of further stroke or palliative status, a serious adverse event (SAE) form should be completed and the participant should be withdrawn from the study. In the event of an unscheduled admission, an adverse event or SAE form should be completed and the principal investigator (PI) should be informed. The PI makes the decision regarding whether the participant should continue to participate or be withdrawn from the study. Participants themselves or their consultees have the right to withdraw from the study at any time, without providing a reason.

The research site team will ensure the accuracy of participant’s address and contact details on file, which may be confirmed using primary and secondary care–linked health records to ensure accuracy. To ensure participant retention, following these checks, a study involvement reminder letter should be sent to the participant’s home address, provided they have been discharged.

#### Period B—90 Days Poststroke (± 14 Days)

At this point, within the study, it is expected that a large proportion of participants will reside in the community. Therefore, participants are to receive study follow-up via either telephone or face-to-face appointment [21]. Telephone appointments should be scheduled with the participant to complete the patient-reported aspect of CRF B via the telephone. Follow-up appointments should be arranged to fall within the 90 days (± 14 days) poststoke window of opportunity, and a maximum of three attempts should be made to contact the participant. In circumstances where three unsuccessful attempts have been made to contact the participant or if the window of opportunity has elapsed, then the participant is to be considered lost to follow-up.

Face-to-face appointments can be used by research sites as a first preference. Participants with either or both communication difficulties and prestroke or poststroke cognitive impairment should be offered the option of either face-to-face or telephone appointments. The face-to-face follow-up appointments should be conducted in accordance with procedures outlined in Period A (≤14 days poststroke) data collection.

If participants are yet to be discharged from the hospital by 90 days (± 14 days) poststroke or have been rehospitalized, for reasons other than further stroke, then participants can also be followed up with CRF B but in line with procedures outlined in Period A (≤14 days poststroke) data collection.

#### 150 Days Poststroke (± 7 Days)

This occurs between Periods B and C at 150 days poststroke (± 7 days). The procedure is identical to that outlined in the 60-day poststroke section of this protocol paper, which should be repeated at the 150-day poststroke (± 7 days) time point.

#### Period C—180 Days Poststroke (± 14 Days)

Data collection at this point is identical to Period B (90 days poststroke). The procedure for this period should be repeated exactly as outlined in the Period B section of this protocol paper using CRF C at the 180-day poststroke (± 14 days) window of opportunity.

#### Long-Term Data Collection

Further funding may be sought to obtain repeat data measurement at future time points, in keeping with the recommendation of the ICHOM Standard Set for Stroke. Currently, there are no defined plans for these types of data collection, but all data will be collected using assessments and outcome measures as outlined in the protocol presented in Figure 2.

### Completion of Assessments, Screens, and Patient-Reported Outcomes

Response options for these data are either complete or incomplete and whether the participant is able or unable to self-complete the relevant PROMs, cognitive screen, and anxiety and depression screen. If the participant is unable to self-complete, then the reason for non–self-completion is to be recorded. Where participants cannot self-complete but are able to give verbal responses, then baseline data collection of PROMs, assessments, and screens are to be administered verbally to ensure that all questions are completed. If the participant is unable to give responses when verbal administration is attempted, the reason for this noncompletion should be recorded on the CRF. Reasons for both non–self-completion and noncompletion of verbal administration should be identified as either potentially cognitive or physical impairment.

#### Cognitive Causes of Noncompletion

Cognitive causes of noncompletion should be recorded on the CRF, and no further attempt at patient-reported data collection should be made at this time. Cognitive causes of noncompletion include, but are not limited to, the following: confusion,drowsiness, history of dementia, aphasia—expressive or receptive, and anosognosia.

#### Physical or Visual Causes of Noncompletion

If the suspected reason for non–self-completion is physical or visual impairment, then the participant is to be provided assistance to complete the PROMs, cognitive screen, and anxiety and depression screen. The administrator should read the question and note down the response. However, there should be no deviation from the wording of the questions or response options. Potential physical or visual deficits that could be expected are as follows: hemiparesis, hemiplegia, hemi-inattention, and hemianopia.

During Periods B and C, if the participant is unable to complete the PROM, cognitive screen, or anxiety and depression screening questions via telephone, a decision about whether a suitable alternative follow-up method is appropriate should be made. This is to be based on clinical judgment, provided the participant still wishes to continue in the study.

During collection of data, the use of secondary sources to aid data collection is permitted for the following; demographic, clinical, lifestyle, poststroke sequelae and functional assessments. The participant’s family, friends, care team, or medical notes are permissible sources of consultation. However, secondary sources are not permitted to aid the completion of the patient-reported data such as cognitive screen, PROM, or anxiety and depression screen.

### Data Analysis

The objective of this study is to estimate the prevalence of morbidity at 6 months with a 95% CI width of ±5%.

The study will be deemed to have ended following the collection of data for Period C from the last participant registered in the study. All participants recruited in the study will be included in the analysis population. All clinical characteristics will be presented in a descriptive narrative.

#### Morbidity Prediction

Baseline clinical and patient demographic variables will be used to predict the mediating effects of a patient exhibiting poststroke morbidity.

Continuous outcomes are analyzed using a mixed-effects linear model, and binary outcomes are analyzed using logistic regression. All estimates will be presented with 95% CI. Sites will be fitted by a random intercept model. For outcomes that analyze at a single time point, we will fit a single random intercept of patients nested within the site using a random intercept model. Moreover, we will be fitting a three-tier multilevel model, including two random intercepts. Multiple patient time points will be fitted within a patient, and the patient will be fitted within a site. Statistical analysis will be performed using Stata Statistical software (StataCorp LLC).

#### Psychometric Analysis

The patient-reported outcomes will be assessed for the following psychometric properties: validity (content validity and convergent validity) and reliability (internal consistency).

#### Missing Outcome Data

Due to the nature of our data collection methods, and evidence from previous studies of this nature, we anticipate negligible missing instrument levels, and in this case, a complete case analysis will be carried out. If the level of missing outcome data is not considered negligible (>5%), missing data will be explored for patterns of missingness and may be imputed using appropriate methods. Imputation methods will depend on the proportion of missingness and reason for the missing data.

### Ethical and Regulatory Considerations

Ethical approval has been granted by an NHS Research Ethics Committee (REC; 18/WA/0299) before recruitment for the study began. The NHS REC has reviewed the study protocol and all relevant trial materials. Where necessary, the REC will review any amendments or alterations to the study design or conduct.

The protocol was developed using the SPIRIT guidelines [20] and a completed SPIRIT checklist is included Multimedia Appendix 1.

#### Amendments

Amendments will be internally reviewed at the coordinating center, and no study amendments will proceed without prior approval of the study sponsor. Amendments that require review by NHS REC will not be implemented until full REC approval has been obtained for the amendment under review and the local participating NHS organization approvals are in place to implement the amendment at the research site. The coordinating center will work with the participating research site to ensure that the necessary arrangements are in place to implement the amendment. The full amendment history of the study protocol will be tracked in the appropriate section of the protocol.

### Data Management

Data will be collected by appropriate health care staff appointed by the PI, and training will be provided by the study team for the delivery and use of assessments required to complete the data collection. A secure online data input and management system is used for all study data. Regular data inspection and quality control will be performed throughout the lifetime of the study. Research sites will retain identifiable study data securely for a minimum of 5 years. Anonymous study data at the study office level will be held for 10 years in line with the sponsor’s requirements. The data custodians will work together to establish a suitable trial data repository for the anonymized study dataset following the conclusion of primary and secondary data analyses.

#### Data Monitoring and Oversight Committee

The Data Monitoring and Oversight Committee (DMC) will convene to oversee the study by providing independent scrutiny of the progress and conduct of the study. The committee consists of a funder’s representative, sponsor’s representative, patient representative/s, and an independent member. The patient’s representative/s will be stroke survivor/s or relative/s or carer/s of a stroke survivor with an interest in stroke care or research. The independent member will be an academic (clinical academic or academic involved in the design and conduct of clinical research) or a suitably qualified health care professional with experience, knowledge, and understanding of the design and conduct of clinical research. Moreover, the independent member will have no institutional affiliation with the chief investigator, coinvestigators, research team at the coordinating center, sponsor, or funder nor will the independent member be involved in the design or conduct of the study. All independent members of the DMC listed previously will have full voting rights.

#### Study Sponsor and Funder

The sponsor, Aneurin Bevan University Health Board, will provide institutional level support for this study. They will ensure safe and proper conduct of the study in line with the International Conference on Harmonization Guideline for Good Clinical Practice and the Declaration of Helsinki [22] and reserve the right to audit all study documents and standard operating procedures at the coordinating center and research sites. The sponsor and the funder (Stroke Implementation Group of the Welsh Government) are entirely independent of the study and have no influence or involvement in the trial design or decision to publish results.

#### Protocol Compliance

Compliance with the protocol and study procedures must be monitored at the research site by the PI, whereas compliance of all study sites will be monitored by the coordinating center, study sponsor, and DMC. Deviations will be monitored by the supplied deviation log, and all deviations must be reported to the study coordinating center within 24 hours of the discovery of the deviation.

All deviations should be reported to the PI at the research site, and all deviation logs are to be ratified by the PI at the research site before they are reported to the coordinating center. The coordinating center will classify the nature of the deviation and will either request the completion of the corrective and preventive action (CAPA) form or, depending on the severity of the deviation, may escalate the deviation to the study sponsor, DMC, local NHS organization research and development department, and national research authority. Adherence to the CAPA outlined in the CAPA form is ultimately the responsibility of the PI. Where deviations from the protocol are found to reoccur, immediate action will be required and such instances could potentially be classified as a serious breach of the protocol or Good Clinical Practice (GCP). Moreover, where deviations are previously the subject of a CAPA, they will be reviewed by the coordinating center, study sponsor, and potentially the DMC. Continued deviations, especially those deviations previously resulting in a CAPA, may be escalated to the local NHS organization’s Research and Development department, which is the national research authority and may result in the suspension of recruitment at the research site.

Serious breaches of GCP or protocol should also be self-referred by the PI at the research site to the appropriate research governance authority, in line with the applicable local and national guidelines.

#### Access to the Final Study Dataset

Access to the final deidentified study dataset will be restricted to the chief investigator, coinvestigators, data manager, and study sponsor. Granting access to the final deidentified study dataset to third parties must be unanimously agreed by the chief investigator and study sponsor. Thus, the chief investigator and the study sponsor will jointly hold the role of data custodians for the study. All requests to access the final study dataset are to be made formally in writing to all those acting as data custodians, whereby all intended analyses are outlined clearly. Acting as a PI this does not grant the individual named as PI at the research site the right to utilize any data arising from the trial. Those PIs wishing to utilize any data resulting from the trial must formally request, in writing, permission to analyze any part of the data arising from the study. Access to the final data will only be granted with the unanimous agreement of the data custodians. Those given access to the final study dataset are only permitted to undertake analyses as outlined in the formal request. A further formal request must be made to the data custodians to adapt, change, or run new analyses not outlined in previous formal requests. The data custodians will work together to establish a suitable trial data repository for the anonymized study dataset following the conclusion of all primary and secondary data analyses

### Consent

#### Informed Consent

All potentially eligible participants will have the capacity to offer valid and informed consent assessed. Potentially eligible participants who do not have the capacity to offer informed consent can also take part in the study through the use of a consultee, thereby ensuring that the study cohort is representative of the poststroke population.

In line with the Mental Capacity Act (2005) England and Wales [23], the potentially eligible participant’s capacity to consent will be presumed unless it is established otherwise. Informed consent will be sought from those potentially eligible participants, where a lack of capacity to consent could not be established as outlined under the Mental Capacity Act (2005) [23].

All potentially eligible participants with the capacity to consent are to be presented with the most current version of the participant information sheet and given a minimum of 24 hours to consider their participation in the study and ask questions or request clarifications. Following this period, written informed consent will be sought from all potential participants with the capacity to consent from the PI or a registered health care professional delegated by the PI.

#### Aphasia or Communication Difficulties

Participants who present with or have a known diagnosis of aphasia or communication difficulties and have the capacity to provide informed consent should be approached using the latest version of the aphasia or communication difficulty–specific participant information sheet.

#### Limb Weakness or Paralysis

If a patient is unable to sign the consent form due to limb weakness or paralysis but has the capacity to provide informed consent, then oral consent will be taken in the presence of a witness, who must not be involved in the study in any capacity. The witness must sign the designated witness consent area on the consent form on behalf of the participant.

#### Consultee

A consultee must be sought when a potential participant who, under the provisions of the Mental Capacity Act (2005) [23], cannot provide informed consent. The consultee is to be provided with the most current version of the consultee information sheet and should be given a minimum of 24 hours to consider the wishes of the potential participant. Following this period of reflection, a written declaration must be obtained from the consultee if he/she believes the potential participant would have no objections to taking part in the study. Those participants who do not regain the capacity to consent are to remain under the consultee declaration for the duration of the study. The consultee has the right to advise the withdrawal of the participant from the study at any time without giving a reason. Moreover, participants have the right to withdraw their participation at any time if they indicate signs of unwillingness to participate.

#### Regaining Capacity to Offer Informed Consent

The loss of capacity to offer informed consent should not be considered as a fixed state, and the assessment of capacity to offer informed consent should not be considered as a final decision to be applied across the whole of the study period. Consultees should be informed that they are to make the study team aware if they believe the participant has regained the capacity to offer informed consent. Participants suspected of regaining capacity to offer informed consent are to be given the most recent version of the regained capacity participant information sheet and 24 hours to consider their continued participation in the study and to ask any questions or clarifications. Subsequently, the participant should be asked to provide informed consent. Alternatively, if a participant wishes to decline continued participation, he/she should be withdrawn from the study. Anonymized data collected prior to withdrawal will be used in the study analysis.

#### Loss of Capacity to Offer Informed Consent

Where it is determined that a participant has lost his/her capacity to offer informed consent, help should be sought from the appointed consultee. Nominated consultees will be made aware of their status as a nominated consultee when the participant initially consents to the study. Therefore, the nominated consultee is to be provided with the appropriate information sheet and given 24 hours to consider if he/she believes that the participant has no objections to continuing to participate in the study. Following the period of reflection, where the consultee believes that the participant would have no objections to continuing in the study, he/she will complete a consultee deceleration form. If the consultee believes that the participant would not want to continue to participate in the study, then the participant should be withdrawn. All anonymized data collection prior to withdrawal will be utilized in the study analysis.

#### Further Contact

All consent and consultee declaration forms include an optional section, which asks participants or consultees to consent to or declare to being contacted at future time points poststroke to seek further consent or consultee declaration to participate in answering longer-term follow-up questions for the study.

#### Confidentiality

Data will be collected and stored in line with the General Data Protection Regulations [24]. Following the receipt of informed consent, each participant will be anonymized and assigned a unique 6-digit Participant Research Number (PRN). Data collected for each participant will be stored alongside their PRN. These tables will not contain any identifiable information from participants. A separate database linking the PRN to the participant’s NHS number will be stored at the research site. This database will be encrypted and restricted to named researchers only under the direct supervision of the PI. Information shared by the research team to the lead researchers outside of the individual research site will be completely anonymized, and lead researchers will only have access to data stored against the PRN. Following the conclusion of the study, the anonymized data will be archived for a period of 10 years in line with the sponsor’s requirements.

## Results

Recruitment opened in October 2018, with 536 participants recruited and 20 sites opened in England and Wales as of September 2019. Patient recruitment was closed on October 2019, with follow-up occurring until April 2020. Data analysis is scheduled to start after all data have been collected, with an aim to publish a peer-reviewed article in late 2020.

## Discussion

This study aims to assess morbidity poststroke using patient-relevant outcome measures. Assessment of morbidity in stroke survivors will indicate the prevalence and type of morbidity to allow for the concentration of support needed for stroke survivors in these areas.

The research team will plan a longer term follow-up of participants of the MORe PREcISE study to explore further changes in morbidity, outcomes, and quality of life.

## Appendix

Multimedia Appendix 1 SPIRIT (Standard Protocol Items: Recommendations for Interventional Trials) checklist.

## Abbreviations

CAPA: corrective and preventative action
CRF: case report form
DMC: Data Monitoring and Oversight Committee
GAD-7: Generalized Anxiety Disorder-7
GCP: Good Clinical Practice
HRQoL: health-related quality of life
ICD: International Classification of Diseases
ICHOM: International Consortium Health Outcomes Measurement
MoCA: Montreal Cognitive Assessment
mRS: Modified Rankin Scale
NHS: National Health Service
PHQ-4: Patient Health Questionnaire-4
PHQ-9: Patient Health Questionnaire-9
PI: principal investigator
PRN: Participant Research Number
PROM: Patient-Reported Outcome Measure
PROMIS-10: Patient-Reported Outcomes Measurement Information System Global Health Short Form-10
REC: Research Ethics Committee
RFA: Rankin Focused Assessment
SF MoCA: Short-Form Montreal Cognitive Assessment
SPIRIT: Standard Protocol Items: Recommendations for Interventional Trials
T-MoCA-Short: Telephone Montreal Cognitive Assessment Short
